# Evaluation of SCD, ACACA and FASN Mutations: Effects on Pork Quality and Other Production Traits in Pigs Selected Based on RNA-Seq Results

**DOI:** 10.3390/ani10010123

**Published:** 2020-01-12

**Authors:** Katarzyna Piórkowska, Martyna Małopolska, Katarzyna Ropka-Molik, Magdalena Szyndler-Nędza, Angelika Wiechniak, Kacper Żukowski, Barry Lambert, Mirosław Tyra

**Affiliations:** 1 Department of Animal Molecular Biology, National Research Institute of Animal Production, Krakowska 1, 32-083 Balice, Poland; katarzyna.piorkowska@izoo.krakow.pl (K.P.); katarzyna.ropka@izoo.krakow.pl (K.R.-M.); 2 Department of Pig Breeding, National Research Institute of Animal Production, Krakowska 1, 32-083 Balice, Poland; magdalena.szyndler@izoo.krakow.pl (M.S.-N.); miroslaw.tyra@izoo.krakow.pl (M.T.); 3 Faculty of Biotechnology and Horticulture, University of Agriculture in Kraków, al. 29 Listopada 54, 31-425 Kraków, Poland; aka.wiechniak@gmail.com; 4 Department of Cattle Breeding, National Research Institute of Animal Production, Krakowska 1, 32-083 Balice, Poland; kacper.zukowski@izoo.krakow.pl; 5 Department of Animal Science and Veterinary Technology, Tarleton State University, Stephenville, TX 76402, USA; blambert@tarleton.edu

**Keywords:** RNA-seq, IMF, selection marker, pig, meat quality

## Abstract

**Simple Summary:**

This study aimed to evaluate mutations within three candidate genes (*SCD, ACACA, FASN*) for their effects on fattening and slaughter characteristics, as well as meat quality traits, including intramuscular fat (IMF) level in pork. They were selected within differentially expressed genes activated in response to variable backfat content obtained using the RNA sequencing method. The RNA-seq analysis identifies mutations/SNPs located in the mRNA and could be a useful tool for prediction of genetic markers in farm animals. The results showed that selection for *FASN* A allele in Polish Large White pigs could lead to improved meat quality traits such as water exudation and meat colour. However, analysed polymorphisms showed only slight effects on fat metabolism and IMF content.

**Abstract:**

In recent years, pig producers have struggled with the problem of low intramuscular fat levels in pork, which impacts palatability and ultimately meat quality. Reduced levels of intramuscular fat are likely the result of breeding objectives aimed at increasing lean meat content. In this study, three mutations within candidate genes for fat content (*SCD, ACACA,* and *FASN)* were selected, based on RNA-seq results and the relationship between polymorphisms in genes related to lipid metabolism, fattening and slaughter characteristics, as well as pork quality, including IMF level, were evaluated to identify selection markers. Moreover, their impact on gene expression was also examined. The PCR–RFLP (polymerase cha- in reaction – restriction fragments length) method was used to establish genotypes and effect sizes of potential genetic markers were estimated using a GLM model. It was identified that a *FASN* missense variant was positively associated with the expression level of this gene, which suggested its linkage with a mutation having a regulatory function. The association study indicated that the FASN missense variant may play a role in the determination of feed conversion and meat colour. In turn, a mutation in the *ACACA* gene showed a relationship with IMF content in the Puławska breed where the differences reached as much as 20%. We suggest considering all three mutations in further studies based on different pig populations due to the crucial role of *SCD, ACACA,* and *FASN* genes in lipid metabolism.

## 1. Introduction

Over the years, linear selection for lean meat content has led to the deterioration of meat quality, including sensory attributes of pork such as flavour, texture and appearance [1,2,3,4]. Due to the high positive correlation between backfat thickness and intramuscular fat (IMF), both were reduced during this selection. Intramuscular fat is responsible for marbling and when present in low quantities results in reduced tenderness, juiciness and firmness [5]. The optimum IMF content has been shown to be in the 2.0–3.4% range [6,7], whereas highly productive breeds are currently characterised by IMF level below 2% [8]. Thus, an increase in IMF will likely lead to improved meat quality. Moreover, IMF content is strongly connected with the physiological and biochemical processes, regulated by genes [9]. Therefore the identification of genetic markers related to IMF would help to improve pig selection and breeding systems. 

To date, the impact of numerous genes related to lipid metabolism and feed intake in the context of affecting fat level have been analysed, including *SCD, ACACA* and *FASN.* The *SCD* gene encodes *stearoyl-CoA desaturase* that plays a crucial role in the conversion of saturated fatty acids (SFAs) into monounsaturated fatty acids (MUFAs). The MUFAs are alleged to be beneficial for health because they aid in reducing low-density lipoprotein cholesterol [10]. Moreover, *SCD* is highly expressed in fat tissue and also in skeletal muscle [11]. Thus, it was proposed as a candidate gene for fatness in livestock and its polymorphisms are frequently considered in research [12]. The study carried out in Berkshire pigs showed that a single nucleotide polymorphism c.*2041T > C in 3′UTR in *SCD* gene region affects fatty acid composition, fat deposition (including IMF) and marbling [13]. In turn, Henriquez-Rodriguez et al. [14] suggested that combined selection for *SCD* T (g.2228T > C) and LEPR C (g.1987C > T) is a good strategy to increase the MUFA/SFA ratio in Duroc pigs. 

The *ACACA* gene encoding *acetyl-coenzyme A carboxylase-α* is also highly important in lipid metabolism, because it catalyses the first committed step in the biosynthesis of long-chain FAs. Gallardo et al. [15] studied variations within porcine *ACACA* gene and analysed their effects on fat content. The authors found two polymorphisms in the coding region (c.4899G > A and c.5196T > C) that influence carcass lean content, IMF and serum HDL-cholesterol concentration. Analysis of Polish pig populations showed that two *ACACA* c.*99T > A and c.*195C > A mutations in the 3′UTR region affected backfat thickness and leanness, including abdominal fat and loin backfat weights, loineye area and meat content of primary cuts [16]. 

The *FASN* gene encodes fatty acid synthase that catalyses the biosynthesis of saturated fatty acids (SFA), mainly palmitic acid from acetyl-CoA and malonyl-CoA [17]. Zappaterra et al. [18] showed that c.265T > C SNP *FASN* polymorphism significantly changes the content of stearic, arachidonic, dihomo-γ-linolenic and arachidonic fatty acid in *longissimus thoracic* of Italian Large White pigs. Grzes et al. [17] found four polymorphisms in the *FASN* gene (c.-2908G > A, c.-2335C > T, c.*42_43insCCCCA and c.*264A > G) which were associated with backfat thickness. Additionally, *FASN* c.-2335C > T influenced cholesterol level in *longissimus thoracic* of Duroc pigs and polyunsaturated fatty acid (PUFA) content in subcutaneous fat tissue of the Pietrain breed. A study conducted in heavy Italian pigs also confirmed the importance of *FASN* gene because its polymorphisms significantly affected quality of heavy pig meat products [19]. 

The present study focused on *FASN, SCD* and *ACACA* polymorphisms identified after transcriptome analysis. The basic aim was to evaluate the effects of potential genetic markers for finishing and slaughter traits, and meat quality parameters including IMF level in Polish pig populations. 

## 2. Materials and Methods 

The research was performed on biological material derived from pigs maintained and slaughtered in the Pig Test Station (PTS, National Research Institute of Animal Production). In the PTS, gilts are slaughtered and dissected. Following carcass evaluation, the meat is intended for consumption. Therefore, our research did not require the approval of the Animal Experimentation Committee.

### 2.1. Animals and Methods

The association analyses were performed on 150 Puławska (PUŁ), 96 Polish Large White (PLW) and 96 Polish Landrace (PL) gilts. Pigs were housed from 30 kg body weight, kept in individual pens and fed ad libitum with two feed mixtures. First, from 30 to 80 kg body weight, the diet contained 13.5 Mj/kg energy, 17–19% crude protein, 2.4–4.0% crude fibre, and second the diet used from 80 to 100 kg body weight, contained 13 Mj/kg energy, 16–18% crude protein and 3.0–5.0% crude fibre, according to Pig Test Station methodology [1]. In the growth period of 30–100 kg animals were weighed once a week and parameter variables such as growth rate (DG; g/day), feed conversation ratio (FC; kg/kg), and daily feed intake (DFI; kg) were measured. Gilts were slaughtered at 100 ± 2 kg body weight in the slaughterhouse located next to (and owned by) the Pig Test Station, and blood samples were collected. Up to 20 min after slaughter, backfat tissue samples were collected from the same place in the carcasses (behind the last rib) and stabilised in RNA-later solution (Ambion, Cambridge, UK). Next, carcasses were chilled (24 h at 4 °C), and then the right half of each carcass was dissected. During the dissection, several carcass characteristics were measured such as loin (LM; kg) and ham mass (HM; kg), average backfat thickness (ABT; cm) from five measurements (backfat thickness over the shoulder blade, at the thoracic part and three measurements at the sacrum part of spine), loin eye area (LEA; cm^2^) between the last thoracic and first lumbar vertebras and carcass yield (%) [20]. Moreover, meat quality traits including meat colour and IMF in the *longissimus dorsi* muscle, and pH in the *longissimus dorsi* and *semimembranosus* muscle were assessed. The pH was measured 45 min and 24 h after slaughter at the last rib according to the PTS procedure and meat colour (redness-a*, yellowness-b* and lightness-L*) was estimated by colorimeter Minolta CR-310. Intramuscular fat was assessed in thawed *longissimus dorsi* homogenates by the Soxhlet method using Soxtherm SOX 406—Gerhardt [21]. Exudation was determined as the amount of free water according to the Grau and Hamm method (filter paper press) [22]. The ratio of pressed water (meat exudate) to total water content was calculated, where 1 cm^2^ of expressed leaked juice ring after pressing corresponds to 10 mg of loose water and total water content is 75% of total meat weight [23]. 

### 2.2. RNA-Sequencing, Differentially Expressed Gene (DEG) and Variant Calling Analyses

The RNA-sequencing analysis included randomly selected gilts (n = 16) from the Puławska breed, which differed in average backfat thickness and showed high and low ABT values. The RNA was isolated by TRI Reagent™ Solution (Ambion) according to the manufacturer’s recommendations. The concentration and quality of the RNA were estimated using TapeStation2200 (Agilent, Palo Alto, CA, USA). The RIN parameter values for all the RNA samples were over 7.5. cDNA libraries were prepared using TruSeq RNA Sample Preparation Kit v2 (Illumina, San Diego, CA, USA) with individual indexing. The sequencing was conducted on a HiScanSQ System (Illumina, San Diego, CA, USA) in single 85 bp cycles using TruSeq Kit v3-HS chemistry (Illumina, San Diego, CA, USA), as described by Piórkowska et al. [24].

The sequence data (GSE122349) has been submitted to the Gene Expression Omnibus (GEO).

Raw read processing was performed as described by Piórkowska et al. [24]. Cleaned reads were represented by Fragments Per Kilobase of transcript per Million mapped reads (FPKM) values. Differentially expressed gene (DEG) analysis was performed using DESeq [25] at a false discovery rate (FDR) ≤ 0.05 and fold-change ≥ 1.5, and functional analysis of DEGs using Panther [26], Classification System v.14 and STRING v.11 (default settings).

Transcript variant identification and functional annotation were done using GATK v. 4.0. [27] and filtering parameters: Fisher Strand (FS) value > 50, QualBy Depth (QD) < 2.0, RMS Mapping Quality (MQ) < 10, Quality < 35, DP < 10 and, splice junction and Variant Effect Predictor using default setting (Ensembl). PROMO [28] v. 8.3 and RegRNA2.0 [29] (default settings) were used to identify transcription factor and miRNA binding sites, respectively. 

### 2.3. Genotyping

Total DNA from whole blood was isolated using Sherlock AX kit (A&A Biotechnology, Gdańsk, Poland) according to the manufacturer’s protocol. Based on differentially expressed gene (DEG) analysis (RNA-seq results GSE122349) and variant calling analysis, three polymorphisms of *FASN, ACACA, SCD*) were chosen for analyses. The PCR-RFLP method was used for genotyping. Primers were designed in Primer3 (v. 0.4.0, default settings) and endonucleases were presented in Table 1. The PCR was performed using AmpliTaq Gold 360 Master Mix (Applied Biosystem™) according to the manufacturer’s protocol. After electrophoresis, band profiles were obtained and are presented below in Table 1.

### 2.4. Statistical Analyses

An ANOVA test (SAS Enterprise v. 7.1 with default settings; SAS Institute, Cary, NC, USA) was used to calculate the differences in gene expression levels (included FPKM values) between gilts with low ABT and high ABT, and with different genotypes.

The General Linear Model (GLM) (SAS Enterprise v. 7.1 with default settings; SAS Institute, Cary, NC, USA) was used for statistical analyses. The linear model for mixed analysis was:

$$Y_{ijkl}=\;\mu +d_{i}+b_{j}+\left(d_{i}\cdot b_{j}\right)+\alpha \left(x_{ijk}\right)+e_{ijkl}\;{\;}_{\;}$$

where: *Y_ijk_*—observation, *µ*—overall mean, *d_i_*—fixed effect of genotype group, *b_j_*—fixed effect of the breed, (*d_i_∙b_j_*)—the interaction between *d_i_* genotype group and breed, *α*(*x_ijk_*)—covariate for weight of the right side of the carcass, *e_ijkl_*—random error. GLM model for analysis within breeds omitted *b_j_*—fixed effect of breed and (*d_i_∙b_j_*)—the interaction between *d_i_* genotype group and breed. The Least Square Means (LSM) method was used for determination of significant differences between genotype groups. The differences in phenotype in particular genotype groups were presented as LSM ± SE.

## 3. Results

### 3.1. Animals

The fattening, carcass and meat quality traits estimated during the test and after slaughter showed significant differences between Puławska pigs and the other two breeds included in our study (Table S1, Supplementary file, please see: https://bit.ly/36JA3vt). 

### 3.2. Potential Genetic Marker Selection

**Figure 1:**
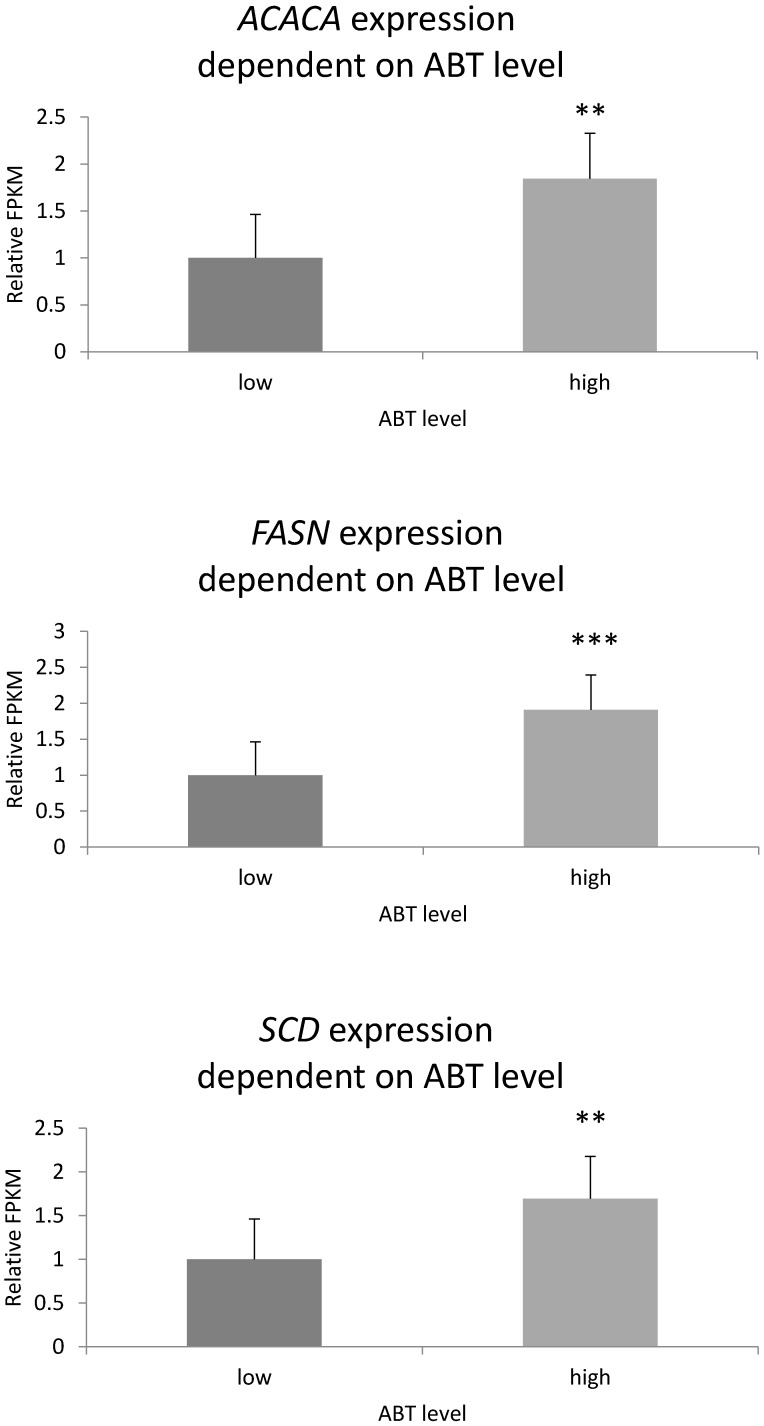
The *FASN*, *SCD*, and *ACACA* expression pattern in fat tissue for various backfat thickness values in the Puławska breed. The expression was estimated based on Fragments Per Kilobase of transcript per Million mapped reads (FPKM). ABT—average backfat thickness.

Pig groups included in RNA-sequencing analysis significantly differed (P < 0.001) in backfat thickness values, where the difference between the low and high backfat thickness groups was 1.59 cm. Differentially expressed gene analysis indicated 450 DEGs associated with backfat thickness (down or up-regulated). The functional analysis of all DEGs found enriched fatty acid biosynthesis processes (FDR < 0.0428) for the *SCD, ACACA, FASN, MGLL* and *PTGES* genes (t.ly/vvqGq). The *SCD, ACACA* and *FASN* were chosen for further analysis. Comparison of the low and high ABT groups indicated significant differences in *FASN, ACACA*, and *SCD* expression level, with higher expression in pigs characterised by thicker backfat (Figure 1).

Variant-calling analysis identified over 150,000 polymorphisms using RNA-seq results, including 229 SNPs within *FASN, SCD*, and *ACACA* genes. Polymorphisms showing minor allele frequency (MAF) over 0.15 within 16 Puławska pigs (included in RNA-seq analysis) and their potential effect on gene expression or protein function were chosen for further study. Among these polymorphisms, three were randomly selected: one missense variant in *FASN*, one 5′UTR variant in *ACACA* and one 3′UTR variant in the *SCD* gene and these were included in further association analysis. Variant-calling and DEG analyses are available at the link t.ly/vvqGq. PROMO analysis identified that the 5′UTR *ACACA* mutation changes binding sites for three transcription factors (Yi, E2F-1 and TOXE), whereas the 3′UTR *SCD* mutation does not affect any miRNA binding sites. 

### 3.3. ACACA, FASN, and SCD Mutation Frequency Analysis

In the *ACACA* gene, two alleles (T and C), and three genotypes (TT, CT and CC) were found in the Puławska breed. The CC genotype was absent in the two other analysed breeds. Similarly, three genotypes in *FASN* and *SCD* genes (AA, AG, GG) were found. In the *FASN* of PLW breed, only AA and AG pigs were observed. Allele and genotype frequencies are shown in Tables S2–S4. 

### 3.4. Effect of FASN c.1328G > A Polymorphism on Pig Production Traits 

The *FASN* gene polymorphism showed a relationship with meat quality characteristics, as well as slaughter and growth traits (Table 2). The muscle of AA pigs (PLW) was characterised by a lighter colour (*p* < 0.05) compared to the other individuals with different genotypes. The pH measured 45 min after slaughter was the lowest in *semimembranosus* muscle of AA PL pigs (*p* < 0.01). 

The AA animals of PLW and PL breeds showed the lowest carcass yield, while AA Puławska pigs presented the highest values of carcass yield. Additionally, in the Polish Large White pigs, the highest weight of loin was noted in GG animals (*p* < 0.01).

Analysis of growth traits showed that AA animals experienced the highest average daily gain and daily feed intake, and the lowest feed conversion (*p* < 0.01). The analysis in particular breeds confirmed this observation with significant results in Puławska pigs, where GG Puławska pigs were characterised by the highest feed conversion (*p* < 0.01) and were the latest to reach the body weight of 100 kg (in the 203rd day of life; *p* < 0.01). The other non-significant differences within genotypes are presented in Table S5.

### 3.5. Effect of ACACA c.-456T > C Polymorphism on Pig Production Traits

The polymorphism in the *ACACA* gene showed a relationship with IMF content, but only in the Puławska breed (*p* < 0.05), where differences between pigs with CC and TT genotypes reached as much as 20% (Table 3). Additionally, the meat of TT Puławska pigs tended to be more yellow compared to the other pigs. In turn, in the Polish Landrace population, an effect of *ACACA* on pH after 45 min was observed, with TT individuals having lower values both in *semimembranosus* and *longissimus dorsi* muscle (*p* < 0.05).

In Puławska pigs, CC animals were characterised by lower carcass yield (*p* < 0.05). In the PL and PLW breeds, the CC genotype was not observed. Puławska gilts had the thinnest backfat (tendency). The average daily gains and daily feed intake were higher in pigs with TT genotype (in the PL and Puławska, tendency) compared to the other gilts. The analysis on all tested breeds confirmed this observation. The other non-significant differences within genotypes are presented in Table S6.

### 3.6. Effect of SCD c.*164A>G Polymorphism on Pig Production Traits

The frequency of AA pigs was very low in all breeds; therefore, these animals were not included in statistical analyses (Table 4). The IMF differed between AG (1.58%) and GG (1.39%) animals (*p* < 0.05), with a similar tendency in Puławska and Polish Landrace pigs in favour of AG animals. Moreover, analysis of meat colour (lightness and yellowness) showed differences between examined genotypes. Lightness was higher in GG pigs, in the analysis independent of the breed effect (*p* < 0.05), and in particular breeds a similar tendency was observed.

In the PL and PLW pigs, carcass yield values were significantly lower in GG animals in comparison to the other individuals, while no differences were noted in Puławska pigs. Moreover, the GG pigs showed higher loin mass in the PL (*p* < 0.05) and higher ham mass in the PLW (*p* < 0.05) populations. The analysis indicates that GG pigs tend to be leaner than AG gilts, due to lower backfat thickness and higher meat content in primary cuts. 

No significant associations were found between the *SCD* mutation and analysed growth traits. The other non-significant differences within genotypes are presented in Table S7.

### 3.7. Gene Expression Level Depending on Genotype

**Figure 2:**
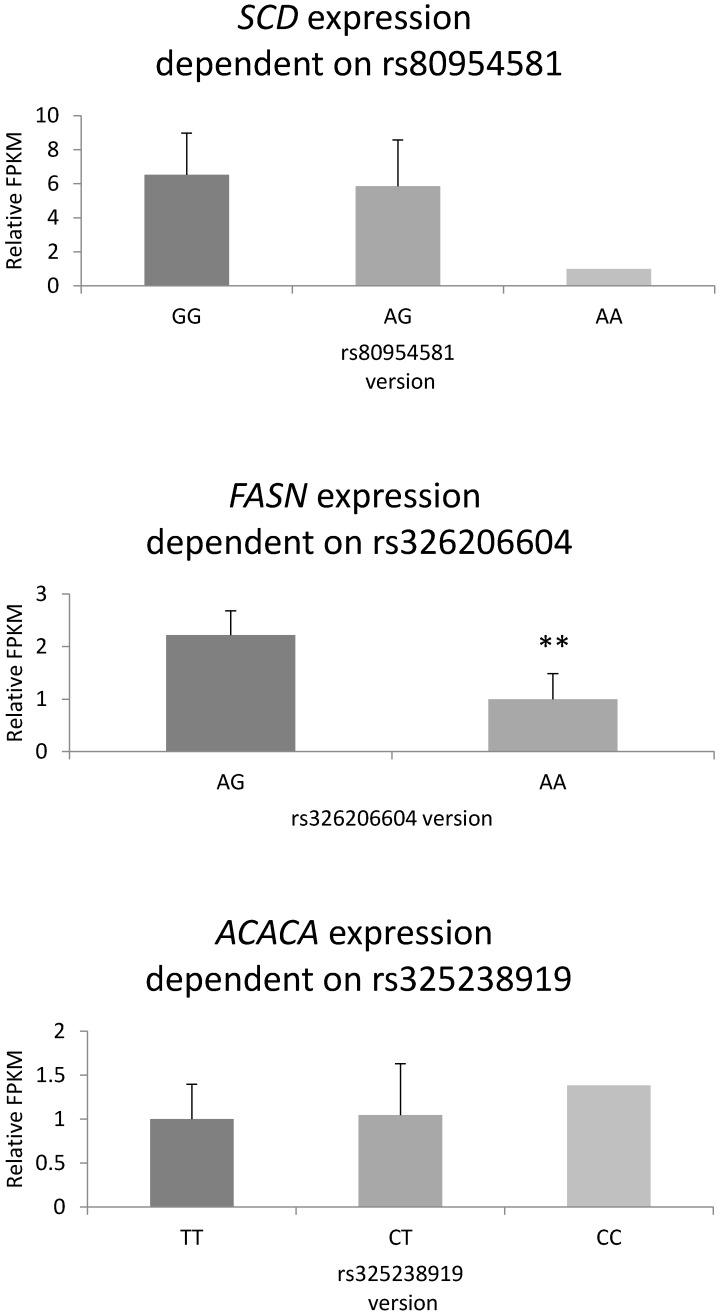
The expression pattern for FASN, SCD, and ACACA genes in backfat tissue depending on their mutation variants for Puławska breed. The expression was estimated based on Fragments Per Kilo Base of exon model per million reads mapped (FPKM).

Based on RNA-seq results (GSE122349) and variant calling, we investigated which of the studied polymorphisms affect gene expression. The results indicated that neither of the mutations in the 3′UTR or 5′UTR regions influence gene expression. Highly significant differences between the AG and GG genotypes of the Arg433Gln FASN missense variant of the *FASN* were observed (Figure 2).

## 4. Discussion

The development of genetic engineering has made available novel methods to improve animal selection and production systems. Given the importance of consumer acceptability, the qualification of genetic markers for economically important traits such an intramuscular fat content should be intensively studied and implemented [20]. In the present study, we analysed three potential genetic markers within genes *FASN, SCD* and *ACACA* strongly associated with lipid metabolism [30]. The investigation included different production traits with particular emphasis on fat content.

The *FASN* mutation is a missense variant G > A, which is located in the coding region of ketoacyl-synthases, C-terminal extension domain, responsible for starting the fatty acid synthesis cycle [31]. This type of mutation changes the amino acid sequence and often disturbs proper protein function. Moreover, it can be also linked with the other causative variants regulating gene expression [32]. Our research showed that the *FASN* c.1328G > A affected the backfat thickness, and AA animals had lower backfat thickness. Gene expression results indicated significantly reduced *FASN* expression in backfat tissue in comparison to AG. It suggests the linkage between this missense variant and another regulatory *FASN* expression mutation. The present *FASN* c.1328G > A polymorphism was not previously examined for its effect, but numerous other polymorphisms of *FASN* were identified and evaluated. Grzes et al. [17] showed an association of *FASN* c.-2335C > T, c.-2908G > A, c.*264A > G polymorphisms with backfat thickness in Polish Large White and Polish Landrace pigs. The authors observed the thickest backfat in CC (c.-2335C > T) and GG (c.-2908G > A and c.*264A > G) homozygotes. Other studies also reported the relationship between *FASN* mutations and backfat thickness [18,33]. In cattle, *FASN* gene polymorphisms were associated with subcutaneous fat and IMF content [34,35]. Authors [34,35] showed that *FASN* g.13232C > T mutation significantly increases IMF level (+7.83%) in Qinchuan cows. Unfortunately, our analysis did not indicate any relation between IMF and *FASN* c.1328G > A mutation. Nevertheless, we observed that IMF content in the analysed breeds was lower than 2% and is not congruent with the optimum content (2–3%), where the consumer acceptability is the highest. Due to strong links between IMF and palatability attributes of meat, consumers created demand for meat with high IMF content [36]. Therefore, identification of genetic markers associated with IMF content is needed.

Classification of pork quality is based on the three main traits: colour, texture and water exudation [37]. Generally, consumers assess meat quality by colour. The standard pork preference is a product that has reddish-pink colour, is firm and non-exudative. Our results showed a relationship between the *FASN* polymorphism and meat colour in the PLW population, where AA pigs were characterised by lighter meat colour. Lightness is related to water holding capacity, which is a significant indicator of meat quality [38]. Another essential meat quality determinant is pH value [39]. The present findings revealed a significant relationship between *FASN* mutation and pH 45 min after slaughter. In the Polish Landrace, heterozygous AG pigs show higher values, both, in *longissimus dorsi* and *semimembranosus* muscles than AA individuals. In addition, the analysis indicated the most exudation in GG pigs, which was related to the lowest pH 45 min after harvest in the Puławska pigs. Similar observation concerns Pale Soft Exudative (PSE) meat, which is characterised by low pH, greater drip cooking losses, and lower marinade uptake [40]. Watanabe et al. [41] suggested that pH is one of the most important factors influencing water holding capacity (WHC), which is important for both consumers and producers [42]. Thus, the polymorphism in the *FASN* gene seems to be promising as a pork quality determinant and should be included in the pool of genetic markers used for selection.

The investigated mutation in the *ACACA* gene is located in the 5′UTR region, which is transcribed but not translated to the amino acid sequence. In turn, the 5′UTR region regulates translation process by forming the complex of secondary structure of the main coding sequence and contains binding sites for miRNAs, transcription factors, and regulatory elements linked to mRNA export [15]. The *ACACA* gene plays an important role in lipid metabolism [15]. According to Ropka-Molik et al. [43], the *ACACA* gene is associated with the fatty acids profile in meat and milk of cattle, sheep and goats. In our results no effect of c.-456T > C mutation on the *ACACA* expression in backfat tissue was showed, if CT and TT pigs were compared. Although PROMO [28] identified that this mutation changes the binding sites for three transcription factors. Nevertheless, the analysed polymorphism can affect *ACACA* expression in different tissues or can play another regulatory role because it forms secondary structures, such as a hairpin loop that regulates the translation process [44]. Moreover, c.-456T > C *ACACA* mutation is located in the upstream region of *TADA2A* gene, which encodes transcriptional adaptor 2A controlling the transcription process. This could be the reason that the present study found that c.-456T > C *ACACA* affected IMF content. In the Puławska breed, the differences between extreme homozygote pigs reached 21%. However, in the PL and PLW breeds, CC pigs were not observed or rare, and TT and CT pigs revealed the same IMF values. The lack of the CC genotype in the PL and PLW populations could be the result of the lean meat selection and is probably linked with the elimination of pigs with low daily gains and low production efficiency. Thus, during the selection process for IMF content, *ACACA* marker is useless in Polish Landrace and Polish Large White breeds. Similarly, Gallardo et al. [15] investigated two mutations in the coding region of *ACACA* gene, and found an association with IMF content in the Duroc breed. In the present study, the investigated *ACACA* 5′UTR mutation affected pH values (both 45 min and 24 h after slaughter) in *semimembranosus* and *longissimus dorsi* muscles of the PL breed. Unfortunately, the effect of *ACACA* mutation on the pH level is not well described in the literature, due to the lack of direct biological association between the acetyl-CoA carboxylase and hydrogen ion release.

The *SCD* gene is involved in the fatty acid biosynthesis and composition of the profile in adipose tissue and animals products (meat and milk) [45]. In our study, *SCD* c.*164A > G mutation did not affect the expression level of *SCD* gene in the adipose tissue. But, it cannot be excluded that it affects *SCD* expression in different tissues. The RegRNA2.0 did not identify any miRNA that is linked to the locus of this mutation. However, the mutations in the 3′UTR regions play other functions during gene expression as an effect on polyadenylation, translation efficiency, localization, and stability of the mRNA [46,47]. Moreover, the mutations in 3′ and 5′untranslated regions are often involved in the regulation of genes expression which are encoded in a neighboring *locus* or are overlapped. The *SCD* c.*164A > G mutation was examined for its effect on fat content and other pig production traits. Our results indicated that the *SCD* c.*164A > G polymorphism affects meat quality traits such as yellowness, lightness and IMF content in analysed pig populations. An increase (13%) in IMF level was found in heterozygotes AG in comparison to GG pigs. Moreover, GG pigs had a darker meat with higher yellow saturation than heterozygotes. Similar tendencies were observed in the Polish Landrace and Puławska breeds. Lim et al. [13] analysed mutation in 3′UTR region of *SCD* (located downstream of ours) found a relation with IMF content in Berkshire pigs. Moreover, *SCD* polymorphisms are recommended as genetic markers for selection to improve fatty acid composition in cattle, [48]. The present *SCD* c.*164A > G contributed also to pork colour such as lightness and yellowness. The yellowness is caused by the deposition of bile pigment (bilirubin), which is the product of heme decomposition and may indicate an excessive breakdown of red blood cells. As a result, the meat is characterised by an unpleasant colour, and odour and may have reduced palatability and consumer acceptance [49]. Another reason for meat yellowing may be the inadequate amount of myoglobin in the muscle, which is associated with an abnormal oxygen exchange into carbon dioxide. However, in the case of raw rabbit meat, the yellow colour is more acceptable to consumers than the red meat colour [50]. Frequency analysis indicates that polymorphism was managed by natural selection because in both native and utility breeds genotype AA was highly rare or absent.

In summary, the identified polymorphisms showed modest, but interesting effects on pig production traits. The *FASN* c.1328G > A missense mutation was probably sensitive for artificial selection conducted in the PL breeds because GG pigs were missing in this breed. However, the selection for *FASN* A allele in the Polish Large White breed could lead to improved meat quality traits such as water exudation and meat colour. The *ACACA* c.-456T > C mutation showed an impact on IMF content, yellowness and daily gains, but only in the Puławska pigs, which belong to native Polish breeds, included to the conservation program and are not under selection pressure. Thus, the usefulness of this marker seems to be low in Polish pig populations used as a maternal component for crossing, where the CC genotype was highly rare or absent. The last investigated mutation *SCD* c.*164A > G located in the 3′UTR region did not resist of natural selection, because AA pigs were missing, independent of analysed breeds. Although statistical analysis was performed only on two genotypes of the *SCD* mutation, we observed a modest effect on exudation and meat colour. Moreover, c.*164A > G *SCD*, affected ham and loin masses, but the observed effect did not have the same direction in particular breeds. Therefore, an approach of individual selection should be applied. We suggest considering all three mutations in further studies, based on commercial pig populations due to the crucial role of *SCD, ACACA*, and *FASN* genes in the lipid metabolism.

## Figures and Tables

**Table 1:** The details of PCR-RFLP method used to identify the polymorphisms in analysed genes.

Gene	SNP	Gene Region	Endonuclease	Digested PCR Product	refSNP	Primers
***SCD***	A/G	3′UTR	*HpyCH4*III	72,46,39/111,46	rs80954581 c.*164A > G	TGCTACCAGGATGCTAAAGATG CAAGAGAAGGACAATGGGACT
***ACACA***	T/C	5′UTR	*Alu*I	126,51,14/101,51, 25,14	rs325238919 c.-456T > C	CTCCCCTCTCTCAGCTCCAG GGCAGGAATGTTACAAAACG
***FASN***	G/A	Missense Arg433Gln	*Hpa*II	208/160	rs326206604 c.1328G > A	CTGACCCCAAGCTCTTTGAC CTCACCTTCGAGGTCGCTCT

**Table 2:** Association of *FASN* gene and analysed traits (LSM ± SE).

Index	Genotype	PLW	PUŁ	PL	Total	GLM		
						*FASN*	Breed	di∙bj
Water exudation (%)	GG	39.8 ± 2.36	28.8 ± 2.50	-	31.6 ± 1.11 ^ab^	*	***	ns
	AG	34.8 ± 1.03	30.6 ± 0.93	35.6 ± 0.89	30.8 ± 0.87 ^a^			
	AA	34.5 ± 2.00	31.2 ± 0.62	36.1 ± 1.04	33.2 ± 1.10 ^b^			
Meat colour L* (lightness)	GG	52.8 ± 0.72 ^a^	54.7 ± 1.51	-	54.9 ± 0.51	ns	***	ns
	AG	53.8 ± 0.31 ^ab^	54.8 ± 0.57	55.5 ± 0.32	54.5 ±0.40			
	AA	54.5 ± 0.31 ^b^	55.2 ± 0.38	55.0 ± 0.38	55.0 ±0.51			
Meat colour b* (yellowness)	GG	2.27 ± 0.27	2.78 ± 0.48	-	3.00 ± 0.16	ns	***	ns
	AG	2.51 ± 0.11	2.85 ± 0.18	2.47 ± 0.09	2.99 ± 0.13			
	AA	2.01 ± 0.23	2.99 ± 0.12	2.45 ± 0.11	2.91 ± 0.16			
pH 45 min (*longissimus dorsi*)	GG	6.35 ± 0.07	6.25 ± 0.08 ^b^	-	6.13 ± 0.03 ^a^	**	***	ns
	AG	6.28 ± 0.05	6.23 ± 0.03 ^ab^	6.34 ± 0.02 ^A^	6.21 ± 0.03 ^ab^			
	AA	6.35 ± 0.06	6.13 ± 0.02 ^a^	6.24 ± 0.02 ^B^	6.17 ± 0.03 ^b^			
pH 45 min (*semimembranosus*)	GG	6.33 ± 0.05	6.21 ± 0.07	-	6.32 ± 0.03	ns	***	ns
	AG	6.34 ± 0.02	6.13 ± 0.03	6.33 ± 0.02^A^	6.34 ± 0.02			
	AA	6.29 ± 0.05	6.12 ± 0.02	6.25 ± 0.02^B^	6.29 ± 0.03			
Carcass yield (%)	GG	77.7 ± 0.36 ^a^	75.1 ± 0.07 ^B^	-	74.8 ± 0.14 ^A^	ns	*	ns
	AG	77.2 ± 0.16 ^ab^	74.3 ± 0.08 ^A^	76.6 ± 0.05 ^A^	75.5 ± 0.11 ^B^			
	AA	76.8 ± 0.31 ^b^	74.5 ± 0.20 ^A^	75.7 ± 0.04 ^B^	75.9 ± 0.14 ^B^			
Average backfat thickness (cm)	GG	1.28 ± 0.09	1.56 ± 0.04	-	1.53 ±0.14 ^A^	ns	*	ns
	AG	1.41 ± 0.04	1.56 ± 0.06	1.20 ± 0.05	1.36 ± 0.11 ^B^			
	AA	1.34 ± 0.08	1.46 ± 0.16	1.24 ± 0.04	1.27 ± 0.14 ^B^			
Loin mass (kg)	GG	6.56 ± 0.14 A	5.06 ± 0.04	-	5.21 ±0.07 ^A^	ns	***	ns
	AG	6.19 ± 0.06 ^AB^	5.08 ± 0.05	5.89 ± 0.07	5.75 ± 0.06 ^B^			
	AA	5.84 ± 0.12 ^B^	5.06 ± 0.11	6.00 ± 0.08	5.91 ± 0.07 ^B^			
Average daily gain (g/day)	GG	919 ± 36	739 ± 58	-	799 ± 21 ^A^	ns	***	ns
	AG	920 ± 16	774 ± 22	958 ± 17	886 ± 16 ^A^			
	AA	955 ± 30	786 ± 14	938 ± 15	925 ± 21 ^B^			
Feed conversion (kg/kg)	GG	2.71 ± 0.07	3.35 ± 0.13 ^B^	-	2.99 ±0.05 ^A^	ns	***	ns
	AG	2.68 ± 0.03	3.06 ± 0.05 ^AB^	2.72 ± 0.03	2.80 ± 0.04 ^B^			
	AA	2.73 ± 0.06	3.01 ± 0.03 ^A^	2.78 ± 0.03	2.81 ± 0.05 ^B^			
Daily feed intake (kg)	GG	2.49 ± 0.10	2.41 ± 0.13	-	2.35 ±0.05 ^A^	ns	***	ns
	AG	2.45 ± 0.04	2.34 ± 0.05	2.60 ± 0.03	2.46 ±0.04 ^A^			
	AA	2.58 ± 0.08	2.34 ± 0.03	2.59 ± 0.04	2.57 ± 0.05 ^B^			
Age at slaughter (day)	GG	165 ± 5.35	203 ± 8.56 ^B^	-	181 ± 3.39 ^A^	ns	***	ns
	AG	169 ± 2.20	182 ± 3.20 ^A^	162 ± 3.22	171 ± 2.71 ^B^			
	AA	162 ± 4.52	183 ± 2.13 ^AB^	167 ± 2.76	169 ± 3.41 ^B^			

Allele: A—wild type; G—mutation; within rows, in each column, means denoted by different letter superscripts differ ^AB^ *p* < 0.01; ^ab^ *p* < 0.05; *** *p* < 0.001, ** *p* < 0.01, * *p* < 0.05. PLW—Polish Large White, PUŁ—Puławska, PL—Polish Landrace. di∙bj—the interaction between dj genotype group and bj breed.

**Table 3:** Association of the *ACACA* mutation and analysed traits (LSM ± SE).

Index	Genotype	PLW	PUŁ	PL	Total	GLM		
						*ACACA*	Breed	di∙bj
IMF (%)	CC	-	1.37 ± 0.15 ^a^	-	1.31 ± 0.10 ^a^	*	***	ns
	CT	1.34 ± 0.09	1.67 ± 0.07 ^b^	1.12 ± 0.02	1.58 ± 0.08 ^b^			
	TT	1.34 ± 0.05	1.72 ± 0.03 ^b^	1.12 ± 0.02	1.59 ± 0.08 ^b^			
Meat colour b* (yellowness)	CC	-	2.50 ± 0.37	-	2.47 ± 0.30	ns	***	ns
	CT	2.40 ± 0.20	2.89 ± 0.16	2.64 ± 0.13	2.73 ± 0.15			
	TT	2.42 ± 0.12	3.05 ± 0.13	2.42 ± 0.09	2.69 ± 0.12			
pH 45 min (*longissimus dorsi*)	CC	-	6.13 ± 0.07	-	6.15 ± 0.06	ns	***	ns
	CT	6.30 ± 0.05	6.14 ± 0.03	6.33 ± 0.03 ^a^	6.22 ± 0.03			
	TT	6.30 ± 0.03	6.17 ± 0.02	6.26 ± 0.02 ^b^	6.23 ± 0.03			
pH 45 min (*semimembranosus*)	CC	-	6.16 ± 0.06	-	6.18 ± 0.05	ns	***	ns
	CT	6.35 ± 0.03	6.11 ± 0.03	6.34 ± 0.02 ^a^	6.21 ± 0.03			
	TT	6.29 ± 0.02	6.13 ± 0.02	6.27 ± 0.02 ^b^	6.23 ± 0.02			
Carcass yield (%)	CC	-	74.1 ± 0.20 ^a^	-	74.8 ± 0.26 ^A^	ns	*	ns
	CT	77.1 ± 0.26	74.5 ± 0.08 ^b^	76.2 ± 0.12	75.5 ± 0.13 ^B^			
	TT	77.4 ± 0.16	74.5 ± 0.07 ^b^	76.3 ± 0.09	75.9 ± 0.11 ^B^			
Average backfat thickness (cm)	CC	-	1.53 ± 0.12	-	1.43 ± 0.10	ns	***	ns
	CT	1.33 ± 0.07	1.58 ± 0.05	1.17 ± 0.05	1.42 ± 0.05			
	TT	1.34 ± 0.04	1.54 ± 0.04	1.25 ± 0.04	1.40 ± 0.04			
Average daily gain (g/day)	CC	-	739 ± 44	-	767 ± 38 ^A^	ns	***	ns
	CT	938 ± 26	776 ± 20	956 ± 20	857 ± 19 ^B^			
	TT	921 ± 15	788 ± 15	961 ± 15	874 ± 16 ^B^			
Daily feed intake (kg)	CC	-	2.23 ± 0.10	-	2.27 ± 0.09 ^a^	ns	***	ns
	CT	2.51 ± 0.07	2.31 ± 0.04	2.58 ± 0.05	2.43 ± 0.04 ^b^			
	TT	2.46 ± 0.04	2.38 ± 0.03	2.63 ± 0.03	2.47 ± 0.04 ^b^			

Allele: C—wild type, T—mutation. Within rows, in each column, means denoted by different letter superscripts differ ^AB^ *p* < 0.01; ^ab^ *p* < 0.05; *** *p* < 0.001, ** *p* < 0.01, * *p* < 0.05. PLW—Polish Large White, PUŁ—Puławska, PL—Polish Landrace. di∙bj —the interaction between dj genotype group and bj breed.

**Table 4:** Association of SCD mutation and analysed traits (LSM ± SE).

Index	Genotype	PLW	PUŁ	PL	Total	GLM		
						*SCD*	Breed	di∙bj
IMF (%)	AA	-	-	-	-	ns	***	ns
	AG	1.28 ± 0.11	1.72 ± 0.08	1.23 ± 0.07	1.58 ± 0.07 ^a^			
	GG	1.36 ± 0.06	1.66 ± 0.06	1.13 ± 0.02	1.39 ± 0.05 ^b^			
Water exudation (%)	AA	-	-	-	-	ns	***	ns
	AG	32.4 ± 2.17	29.9 ± 0.91	35.2 ± 2.44	30.9 ± 1.18			
	GG	35.6 ± 1.16	30.6 ± 0.64	35.8 ± 0.76	33.5 ± 0.93			
Meat colour L* (lightness)	AA	-	-	-	-	*	ns	ns
	AG	53.1 ± 0.60	54.5 ± 0.54	54.5 ± 0.89	54.3 ± 0.55 ^a^			
	GG	54.1 ± 0.32	55.6 ± 0.38	55.1 ± 0.28	55.1 ± 0.43 ^b^			
Meat colour b* (yellowness)	AA	-	-	-	-	*	***	ns
	AG	2.36 ± 0.24	2.69 ± 0.17 ^a^	2.11 ± 0.25	2.57 ± 0.18 ^a^			
	GG	2.37 ± 0.13	3.11 ± 0.12 ^b^	2.43 ± 0.08	2.71 ± 0.14 ^b^			
pH 45 min (*longissimus dorsi*)	AA	-	-	-	-	ns	***	ns
	AG	6.31 ± 0.06	6.17 ± 0.03	6.25± 0.06	6.21± 0.04			
	GG	6.29 ± 0.03	6.14 ± 0.02	6.30± 0.02	6.19± 0.03			
Loin mass (kg)	AA	-	-	-	-	ns	***	ns
	AG	6.39 ± 0.16^a^	5.00 ± 0.05	5.64 ± 0.17 ^a^	5.32 ± 0.08 ^a^			
	GG	6.09 ± 0.08^b^	5.06 ± 0.04	6.03 ± 0.05 ^b^	5.64 ± 0.06 ^b^			
Ham mass (kg)	AA	-	-	-	-	ns	***	ns
	AG	9.05 ± 0.16^a^	8.72 ± 0.08	9.28 ± 0.13	8.84 ± 0.09 ^a^			
	GG	9.36 ± 0.09^b^	8.80 ± 0.05	9.26 ± 0.04	9.09 ± 0.07 ^b^			

Allele: A—wild type, G—mutation. Within rows, in each column, means denoted by different letter superscripts differ ^AB^ *p* < 0.01; ^ab^ *p* < 0.05; ^***^p < 0.001, ^**^p < 0.01, ^*^ *p* < 0.05. PLW—Polish Large White, PUŁ—Puławska, PL—Polish Landrace. di∙bj—the interaction between dj genotype group and bj breed.

## Supplementary Materials

The following are available online at https://www.mdpi.com/2076-2615/10/1/123/s1, Table S1: Pig trait values dependent on breed effect (mean ± S.D). Table S2: The frequencies of genotypes and alleles in *FASN* gene. Table S3: The frequencies of genotypes and alleles in *SCD* gene. Table S4: The frequencies of genotypes and alleles in *ACACA* gene. Table S5: Association of FASN mutation and analysed traits (LSM ± S.E.). Table S6: Association of the ACACA mutation and analysed traits (LSM ± S.E). Table S7: Association of SCD mutation and analysed traits (LSM ± S.E).
