# Supplementary material for: Evaluation of SCD, ACACA and FASN Mutations: Effects on Pork Quality and Other Production Traits in Pigs Selected Based on RNA-Seq Results
Source: Animals (Basel). 2020 Jan 12;10(1):123. doi: 10.3390/ani10010123 (PMC7023423; doi:10.3390/ani10010123)
Supplement: Supplementary file 1 [file animals-10-00123-s001.pdf]

Table S1. Pig trait values dependent on breed effect (mean±S.D).

Index	PLW (96)	PUŁ (150)	PL (96)	Total (336)
**Water exudation (%)**	35.6 ± 7.6 ^{A}	28.8 ± 2.50 ^{B}	36.2 ± 6.54 ^{A}	33.4 ± 0.44
**Meat color L*(lightness)**	53.8 ± 2.43 ^{A}	55.1 ± 3.73 ^{B}	55.1 ± 2.31 ^{B}	54.8 ± 3.10
**Meat color b*(yellowness)**	2.37 ± 0.91 ^{A}	2.94 ± 1.17 ^{B}	2.45 ± 0.68 ^{A}	2.65 ± 1.02
**pH 45 min (*longissimus dorsi*)**	6.30 ± 0.19 ^{A}	6.16 ± 0.21 ^{B}	6.28 ± 0.16 ^{A}	6.23 ± 0.20
**pH 24 h (*longissimus dorsi*)**	5.59 ± 0.11 ^{A}	5.52 ± 0.09 ^{B}	5.61 ± 0.10 ^{A}	5.57 ± 0.11
**pH 45 min (*semimembranosus*)**	6.33 ± 0.16 ^{A}	6.21 ± 0.07 ^{B}	6.28 ± 0.14 ^{A}	6.22 ± 0.19
**pH 24 h (*semimembranosus*)**	5.62 ± 0.12 ^{A}	5.56 ± 0.11 ^{B}	5.65 ± 0.10 ^{A}	5.60 ± 0.11
**IMF (%)**	1.34 ± 0.41 ^{A}	1.70 ± 0.46 ^{B}	1.12 ± 0.14 ^{C}	1.41 ± 0.43
**Carcass yield (%)**	77.2 ± 2.71 ^{A}	74.5 ± 2.28 ^{B}	76.2 ± 2.27 ^{C}	75.7 ± 2.47
**Lion mass (kg)**	6.18 ± 0.58 ^{A}	5.06 ± 0.43 ^{B}	5.95 ± 0.60 ^{C}	5.62 ± 0.73
**Ham mass (kg)**	9.28 ± 0.62 ^{A}	8.78 ± 0.62 ^{B}	9.23 ± 0.40 ^{A}	9.05 ± 0.61
**Average backfat thickness (cm)**	1.31 ± 0.31 ^{Aa}	1.55 ± 0.42 ^{B}	1.23 ± 0.28 ^{Ab}	1.40 ± 0.38
**Loin eye area (cm^2)**	52.2 ± 5.37	51.9 ± 5.53	53.4 ± 4.50	52.4 ± 5.22
**Lean meat percentage (%)**	61.0 ± 3.01^{Aa}	60.1 ± 3.08 ^{Ab}	62.4 ± 2.21 ^{B}	61.1 ± 2.95
**Primary cuts (kg)**	24.1 ± 1.27 ^{A}	22.2 ± 1.45 ^{B}	24.0 ± 1.09 ^{A}	23.2 ± 1.57
**Average daily gain (g/day)**	924 ± 107 ^{A}	778 ± 141 ^{B}	945 ± 105 ^{A}	865 ± 146
**Feed conversion (kg/kg)**	2.69 ± 0.22 ^{A}	3.04 ± 0.33 ^{B}	2.75 ± 0.20 ^{A}	2.86 ± 0.32
**Daily feed intake (kg)**	2.48 ± 0.30 ^{A}	2.33 ± 0.31 ^{B}	2.59 ± 0.25 ^{C}	2.44 ± 0.31
**Age at slaughter (day)**	167 ± 16.4 ^{A}	184 ± 21.2 ^{B}	165 ± 19.9 ^{A}	174 ± 22.6

Mean± standard deviation. Within rows, in each column, means denoted by different letter superscripts differ ^{AB}P<0.01; ^{ab}P<0.05. PLW – Polish Large White, PUŁ – Puławska, PL – Polish Landrace.

Table S2. The frequencies of genotypes and alleles in *FASN* gene.

	Genotype			Alleles	
	GG	AG	AA	G	A
PL	-	0,42 (39)	0,58 (54)	0,21	0,79
PLW	0,11 (10)	0,73 (65)	0,16 (14)	0,48	0,52
PUŁ	0,66 (100)	0,3 (46)	0,04 (6)	0,81	0,19

Table S3. The frequencies of genotypes and alleles in *SCD* gene.

	Genotype			Alleles	
	GG	AG	AA	G	A
PL	0,91 (74)	0,09 (7)	-	0,96	0,04
PLW	0,82 (55)	0,18 (12)	-	0,91	0,09
PUŁ	0,66 (99)	0,32 (49)	0,02 (3)	0,82	0,18

Table S4. The frequencies of genotypes and alleles in *ACACA* gene.

	Genotype			Alleles	
	TT	CT	CC	T	C
PL	0,62 (49)	0,35 (28)	0,03 (2)	0,80	0,20
PLW	0,74 (64)	0,24 (21)	0,01 (1)	0,87	0,13
PUŁ	0,57 (87)	0,36 (55)	0,07 (10)	0,75	0,25

Table S5. Association of *FASN* mutation and analyzed traits (LSM±S.E.).

Index	Genotype	PLW	PUŁ	PL	Total	GLM	
						FASN	Breed
**IMF (%)**	GG	1.36±0.11	1.66±0.05	-	1.56±0.06	ns	***
	AG	1.31±0.05	1.75±0.08	1.11±0.02	1.58±0.05		
	AA	1.44±0.11	1.52±0.19	1.15±0.02	1.56±0.07		
**Meat color a* (redness)**	GG	16.0±0.49	15.8±0.11	-	16.8±0.20	ns	ns
	AG	16.8±0.27	15.7±0.14	16.3±0.14	16.7±0.16		
	AA	17.0±0.42	15.9±0.32	16.5±0.16	16.6±0.18		
**pH 24 h (*longissimus dorsi*)**	GG	5.61±0.03	5.52±0.01	-	5.49±0.02	ns	***
	AG	5.59±0.01	5.53±0.01	5.61±0.01	5.49±0.01		
	AA	5.59±0.03	5.52±0.04	5.61±0.02	5.49±0.02		
**pH 24 h (*semiembranosus*)**	GG	5.62±0.04	5.57±0.04	-	5.60±0.02	ns	***
	AG	5.63±0.02	5.56±0.02	5.65±0.01^{a}	5.59±0.02		
	AA	5.61±0.04	5.53±0.01	5.65±0.02^{b}	5.58±0.02		
**Ham mass (kg)**	GG	9.30±0.18	8.80±0.16	-	8.84±0.08^{A}	ns	***
	AG	9.21±0.08	8.72±0.07	9.18±0.06	9.05±0.07^{B}		
	AA	9.27±0.15	8.96±0.06	9.29±0.05	9.26±0.08^{C}		
**Loin eye area (cm^2)**	GG	52.4 ± 1.60	51.9 ± 1.63	-	51.9 ± 0.80	ns	***
	AG	52.6 ± 0.70	51.9 ± 0.71	53.2 ± 0.6	52.5 ± 0.63		
	AA	49.3 ± 1.36	54.6 ± 0.56	53.2 ± 0.5	52.3 ± 0.80		
**Lean meat percentage (%)**	GG	61.8 ± 0.86	60.3 ± 0.98	-	60.4 ± 0.47^{A}	ns	***
	AG	60.9 ± 0.37	60.0 ± 0.43	62.4 ± 0.34	61.0 ± 0.37^{AB}		
	AA	61.0 ± 0.73	61.0 ± 0.34	62.1 ± 0.30	61.8 ± 0.47^{B}		
**Primary cuts (kg)**	GG	24.4 ± 0.34	22.3 ± 0.35	-	22.5 ± 0.17^{A}	ns	***
	AG	24.0 ± 0.15	22.1 ± 0.15	23.8 ± 0.13	23.3 ± 0.14^{B}		
	AA	23.7 ± 0.29	22.9 ±0.112	24.0 ± 0.12	23.9 ± 0.17^{C}		

Allele: A– wild type; G– mutation; Within rows, in each column, means denoted by different letter superscripts differ ^{AB}P<0.01; ^{ab}P<0.05. PLW – Polish Large White, PUŁ – Puławska, PL – Polish The linear model for mixed analysis was: $\boldsymbol{Y_{ijkl} = \mu + d_i + b_i + (d_i \cdot b_i) + \alpha(x_{ijk}) + e_{jikl}}$ where: Y_{ijk} – observation, μ – overall mean, d_i –fixed effect of genotype group, b_j – fixed effect of the breed, $(d_i{\cdot}b_j)$ – the interaction between d_i genotype group and breed, $\alpha(x_{ijk})$ – covariate for weight of the right side of the carcass, e_{ijkl} – random error. GLM model for analysis within breeds omitted b_j – fixed effect of breed and $(d_i{\cdot}b_j)$ – the interaction between d_i genotype group and breed.Landrace.

Table S6. Association of the *ACACA* mutation and analyzed traits (LSM±S.E).

Index	Genotype	PLW	PUŁ	PL	Total	GLM *ACACA*	GLM Breed
**Water exudation (%)**	CC	-	30.7 ± 1.97	-	30.9 ±2.02		
	CT	37.3 ± 1.76	30.6 ± 0.85	36.2 ±1.29	33.5 ±0.99	ns	**
	TT	34.9 ± 1.04	29.9 ± 0.68	36.8 ±0.96	33.2 ± 0.84		
**Meat color L* (lightness)**	CC	-	54.6 ± 1.18	-	54.7 ± 0.87		
	CT	53.7 ± 0.52	55.3 ± 051	55.5 ±0.44	55.1 ± 0.69	ns	***
	TT	54.1 ± 0.31	55.1 ± 0.41	55.0 ±0.33	54.7 ± 0.66		
**Meat colour a* (redness)**	CC	-	15.7 ± 0.32	-	15.9 ± 0.35		
	CT	16.7 ± 0.35	15.7 ± 0.14	16.3 ± .19	16.1 ± 0.17	ns	ns
	TT	16.7 ± 0.21	15.8 ± 0.11	16.5 ±0.14	16.3 ± 0.15		
**pH 24 h (*longissimus dorsi*)**	CC	-	5.54 ± 0.03	-	5.56 ± 0.03	ns	***
	CT	5.62 ± 0.02	5.52 ± 0.01	5.63 ± 0.02	5.57 ± 0.02		
	TT	5.65 ± 0.01	5.53 ± 0.01	5.60 ± 0.02	5.56 ± 0.02		
**pH 24 h (*semimembranosus*)**	CC	-	5.58 ± 0.03	-	5.59 ± 0.03	ns	***
	CT	5.64 ± 0.03	5.55 ± 0.01	5.68 ± 0.02[a]	5.60 ± 0.02		
	TT	5.62 ± 0.02	5.57 ± 0.01	5.63 ± 0.02[b]	5.60 ± 0.02		
**Loin mass (kg)**	CC	-	5.06 ± 0.11	-	5.41 ± 0.13[a]		
	CT	6.10 ± 0.11	5.08 ± 0.05	6.03 ± 0.09	5.54 ± 0.06[ab]	ns	***
	TT	6.25 ± 0.06	5.06 ± 0.04	5.90 ± 0.07	5.65 ± 0.05[b]		
**Ham mass (kg)**	CC	-	8.80 ± 0.16	-	8.92 ± 0.15		
	CT	9.37 ± 0.13	8.73 ± 0.07	9.28 ± 0.09	9.00 ± 0.07	ns	***
	TT	9.22 ± 0.08	8.80 ± 0.06	9.26 ± 0.07	9.05 ± 0.06		
**Loin eye area (cm^2)**	CC	-	52.9 ± 1.63	-	53.7 ± 1.45		
	CT	51.4 ± 1.20	52.4 ± 0.71	53.6 ± 0.7	52.5 ± 0.71	ns	***
	TT	52.4 ± 0.71	51.6 ± 0.56	53.3 ± 0.3	52.3 ± 0.61		
**Lean meat percentage (%)**	CC	-	61.0 ± 0.98	-	61.5 ± 0.84		
	CT	61.7 ± 0.63	59.8 ± 0.43	62.4 ± 0.39	60.9 ± 0.41	ns	***
	TT	60.8 ± 0.37	60.4 ± 0.34	62.3 ± 0.29	61.0 ± 0.35		
**Primary cuts (kg)**	CC	-	22.4 ± 0.35	-	22.9 ± 0.31		
	CT	24.1 ± 0.25	22.1 ± 0.15	24.0 ± 0.14	23.0 ± 0.15	ns	***
	TT	24.1 ± 0.15	22.3 ± 0.112	23.9 ± 0.11	23.3 ± 0.13		
**Feed conversion (kg/kg)**	*CC*	-	3.03 ± 0.10	-	2.98 ± 0.08		
	CT	2.68 ± 0.05	3.02 ± 0.05	2.71 ± 0.04	2.87 ± 0.04	ns	***
	TT	2.67 ± 0.03	3.06 ± 0.04	2.75 ± 0.03	2.86 ± 0.03		
**Age at slaughter (day)**	*CC*	-	188 ± 7	-	182 ± 6[a]		
	CT	165 ± 4	185 ± 3	166 ± 4	176 ± 3[ab]	ns	***
	TT	168 ± 2	183 ± 2	162 ± 3	173 ± 3[b]		

Allele: C – wild type, T – mutation. Within rows, in each column, means denoted by different letter superscripts differ [AB]P<0.01; [ab]P<0.05. PLW – Polish Large White, PUŁ – Puławska, PL – Polish Landrace. The linear model for mixed analysis was: $\boldsymbol{Y_{ijkl}} = \boldsymbol{\mu} + \boldsymbol{d_i} + \boldsymbol{b_i} + (\boldsymbol{d_i} \cdot \boldsymbol{b_i}) + \boldsymbol{\alpha}(\boldsymbol{x_{ijk}}) + \boldsymbol{e_{jikl}}$ where: Y_{ijk} – observation, μ – overall mean, d_i –fixed effect of genotype group, b_j – fixed effect of the breed, ($d_i \cdot b_j$) –the interaction between d_i genotype group and breed, $\alpha(x_{ijk})$ – covariate for

weight of the right side of the carcass, e_{ijkl} – random error. GLM model for analysis within breeds omitted b_j – fixed effect of breed and $(d_i \cdot b_j)$ – the interaction between d_i genotype group and breed.

Table S7. Association of SCD mutation and analyzed traits (LSM±S.E).

Index	Genotype	PLW	PUŁ	PL	Total	GLM *SCD*	GLM Breed
**Meat colour a* (redness)**	AA	-	-	-	-	ns	*
	AG	16.4 ± 0.43	15.9 ± 0.15	16.2 ± 0.39	16.0 ± 0.21		
	GG	16.7 ± 0.23	15.7 ± 0.11	16.4 ± 0.12	16.2 ± 0.16		
**pH 24 h (longissimus dorsi)**	AA	-	-	-	-	ns	***
	AG	5.62 ± 0.03	5.52 ± 0.01	5.68 ± 0.04	5.55 ± 0.02		
	GG	5.59 ± 0.02	5.52 ± 0.01	5.61 ± 0.01	5.57 ± 0.02		
**pH 45 min (semimembranosus)**	AA	-	-	-	-	ns	***
	AG	6.32 ± 0.05	6.13 ± 0.03	6.27 ± 0.05	6.18 ± 0.03		
	GG	6.32 ± 0.03	6.12 ± 0.02	6.29 ± 0.02	6.22 ± 0.03		
**pH 24 h (semimembranosus)**	AA	-	-	-	-	ns	***
	AG	5.67 ± 0.04	5.56 ± 0.02	5.68 ± 0.04	5.59 ± 0.02		
	GG	5.63 ± 0.02	5.56 ± 0.01	5.65 ± 0.01	5.61 ± 0.02		
**Carcass yield (%)**	AA	-	-	-	-	ns	**
	AG	77.7±0.33^{a}	74.2±0.09	77.1±0.24^{A}	75.2±0.15		
	GG	77.1±0.18^{b}	74.6±0.06	76.3±0.07^{B}	75.8±0.12		
**Average backfat thickness (cm)**	AA	-	-	-	-	ns	***
	AG	1.39 ± 0.08	1.55 ± 0.06	1.35 ± 0.10	1.50 ± 0.06		
	GG	1.30 ± 0.04	1.56 ± 0.04	1.23 ± 0.03	1.39 ± 0.05		
**Loin eye area (cm^{2})**	AA	-	-	-	-	ns	***
	AG	52.4 ± 1.42	51.1 ± 0.74	52.9 ± 1.54	51.6 ± 0.86		
	GG	52.0 ± 0.76	52.2 ± 0.52	53.4 ± 0.47	52.5 ± 0.68		
**Lean meat percentage (%)**	AA	-	-	-	-	ns	***
	AG	59.9 ± 0.79	59.9 ± 0.46	61.4 ± 0.77	60.0 ± 0.50^{a}		
	GG	61.5 ± 0.42	60.4 ± 0.32	62.4 ± 0.24	61.3 ± 0.37^{b}		
**Primary cuts (kg)**	AA	-	-	-	-	ns	***
	AG	23.9 ± 0.31	22.0 ± 0.16	23.8 ± 0.30	22.5 ± 0.18^{a}		
	GG	24.2 ± 0.17	22.3 ± 0.11	24.0 ± 0.09	23.3 ± 0.15^{b}		
**Average daily gain (g/day)**	GG	-	-	-	-	ns	***
	AG	967±32	778 ± 21	906 ± 41	826 ± 23		
	AA	926±17	779 ± 15	948 ± 13	870 ± 18		
**Feed conversion (kg/kg b. w.)**	GG	-	-	-	-	ns	***
	AG	2.62 ± 0.07	2.99 ± 0.05	2.79 ± 0.08	2.90 ± 0.05		
	AA	2.68 ± 0.04	3.08 ± 0.03	2.76 ± 0.02	2.93 ± 0.03		
**Daily feed intake (kg)**	GG	-	-	-	-	ns	***
	AG	2.54 ± 0.10	2.31 ± 0.05	2.52 ± 0.10	2.37 ± 0.05		
	AA	2.48 ± 0.05	2.37 ± 0.03	2.60 ± 0.03	2.47 ± 0.04		
**Age at slaughter (day)**	GG	-	-	-	-	ns	***
	AG	163 ± 5	184 ± 3	159 ± 3	177 ± 4		
	AA	166 ± 3	184 ± 9	166 ± 2	174 ± 3		

Allele: A– wild type, G – mutation. Within rows, in each column, means denoted by different letter superscripts differ ^{AB}P<0.01; ^{ab}P<0.05. PLW – Polish Large White, PUŁ – Puławska, PL – Polish Landrace. The linear model for mixed analysis was: $\boldsymbol{Y_{ijkl} = \mu + d_i + b_i + (d_i \cdot b_i) + \alpha(x_{ijk}) + e_{jikl}}$ where: Y_{ijk} – observation, μ – overall mean, d_i –fixed effect of genotype group, b_j – fixed effect of the breed, ($d_i \cdot b_j$) –the interaction between d_i genotype group

and breed, $\alpha(x_{ijk})$ – covariate for weight of the right side of the carcass, e_{ijkl} – random error. GLM model for analysis within breeds omitted b_j – fixed effect of breed and $(d_i \cdot b_j)$ – the interaction between d_i genotype group and breed.
